# Identification and genome analysis of a novel picornavirus from captive belugas (*Delphinapterus leucas*) in China

**DOI:** 10.1038/s41598-021-00605-y

**Published:** 2021-10-25

**Authors:** Gaoyu Wang, Yi Huang, Weijia Zhang, Ruoyan Peng, Jun Luo, Sisi Liu, Shijie Bai, Xiaoyuan Hu, Zhiqiang Wu, Fan Yang, Shu Shen, Yun Zhang, Chuanning Tang, Xiuji Cui, Lina Niu, Gang Lu, Songhai Li, Fei Deng, Peijun Zhang, Jiang Du, Feifei Yin

**Affiliations:** 1 Key Laboratory of Tropical Translational Medicine of Ministry of Education, Hainan Medical University, Haikou, 571199 China; 2 Institute of Deep-Sea Science and Engineering, Chinese Academy of Sciences, Beijing, China; 3 Hainan Medical University, The University of Hong Kong Joint Laboratory of Tropical Infectious Diseases, Hainan Medical University, Haikou, 571199 China; 4 Department of Pathogen Biology, Hainan Medical University, Haikou, 571199 China; 5 NHC Key Laboratory of Systems Biology of Pathogens, Institute of Pathogen Biology, Chinese Academy of Medical Sciences, Peking Union Medical College, Beijing, 100005 China; 6 Dalian Sun Asia Tourism Holding Co. Ltd., Dalian, 116023 China; 7 Qingdao Polar Haichang Ocean Park, Qingdao, 266003 China; 8 State Key Laboratory of Virology and National Virus Resource Center, Wuhan Institute of Virology, Chinese Academy of Sciences, Wuhan, China

**Keywords:** Evolutionary genetics, Microbial genetics, Viral genetics, Virology, Metagenomics, Viral evolution, Microbiology

## Abstract

The discovery of new viruses is important for predicting their potential threats to the health of humans and other animals. A novel picornavirus was identified from oral, throat, and anal swab samples collected from belugas (*Delphinapterus leucas*), from Dalian Sun Asia Tourism Holding Co., China, between January and December 2018, using a metagenomics approach. The genome of this novel PicoV-HMU-1 strain was 8197 nucleotides (nt) in length, with a open reading frame (from 1091 to 8074 nt) that encoded a polyprotein precursor of 2328 amino acids. Moreover, the genomic length and GC content of PicoV-HMU-1 were within the ranges found in other picornaviruses, and the genome organization was also similar. Nevertheless, PicoV-HMU-1 had a lower amino acid identity and distinct host species compared with other members of the Picornaviridae family. Phylogenetic trees were constructed based on the P1 and 3D amino acid sequences of PicoV-HMU-1 along with representative members of the Picornaviridae family, which showed that PicoV-HMU-1 was related to unclassified bat picornaviruses groups. These findings suggest that the PicoV-HMU-1 strain represents a potentially novel genus of picornavirus. These data can enhance our understanding of the picornavirus genetic diversity and evolution.

## Introduction

Members of the family *Picornaviridae* are small, icosahedral, non-enveloped, positive-sense ( +) single-stranded RNA (ssRNA) viruses that can cause various symptoms, ranging from mild febrile illness to severe diseases of the respiratory, cardiac, hepatic, mucocutaneous, and central nervous systems^1^. According to the International Committee on Taxonomy of Viruses (https://talk.ictvonline.org/), the family *Picornaviridae*, order *Picornavirales*, currently consists of 158 species grouped into 68 genera, which include the genera *Aphthovirus* (foot-and-mouth disease virus), *Cardiovirus* (Cardiovirus A), *Enterovirus* (Enterovirus A and Rhinovirus A), *Hepatovirus* (Hepatovirus A), *Limnipivirus* (Limnipivirus A), and *Parechovirus* (Human parechovirus, Ljungan virus, and Sebokele virus).

Picornaviruses belong to the family *Picornaviridae* (https://www.ictv.global/report/picornaviridae) and have a genome that is 6.7–10.1 kilobases in size and contain a single open reading frame (ORF) that encodes a large polyprotein that is cleaved into structural and non-structural proteins by viral proteases. One exception is the genus *Dicipivirus*, which possesses two separated ORFs^2^. The characteristic organizational pattern of the picornaviral genome is VPg–5′-UTR–(L)–P1–P2–P3–3′-UTR–poly(A)^2^. The 5′-untranslated region (UTR) of picornaviruses is a structured functional region that contains an internal ribosomal entry site (IRES), which mediates the end-independent initiation of translation^3^. The single ORF encodes a polyprotein precursor that is divided into three regions (P1, P2, and P3), of which P1 encodes the capsid proteins, whereas P2 and P3 encode proteins involved in proteolytic processing and viral replication. The P1 region is derived from VP4 to VP1, the P2 region is derived from 2A to 2C, and the P3 region is derived from 3A to 3D^1^.

Advances in metagenomics have allowed the identification of new viral genomic sequences with clinical and epidemiological value. Metagenomic technology has facilitated virus discovery due to the enhanced capacity to detect viruses with an unknown genetic background. Recently, several novel picornaviruses were discovered within different hosts, including a novel picornavirus from *Macaca mulatta* in China identified by viral metagenomic analysis^4^. Furthermore, a novel bovine picornavirus was discovered in Japan^5^, and several picornaviruses, including three bat, one feline, and one canine picornaviruses were discovered in Hong Kong^6–8^. The discovery of novel picornavirus provides the foundation for virus traceability and evolutionary research. Overall, approximately 75% of emerging diseases are estimated to arise from zoonotic sources^9^. Zoological parks and aquariums provide a unique opportunity for emerging virus surveillance. The discovery of novel viruses may help predict their potential threat to the health of humans and other animals. In this report, we analyzed by high-throughput next-generation sequencing (NGS) a novel picornavirus identified in belugas (*Delphinapterus leucas*) from an aquarium.

## Results

### Metagenomic analysis

**Figure 1:**
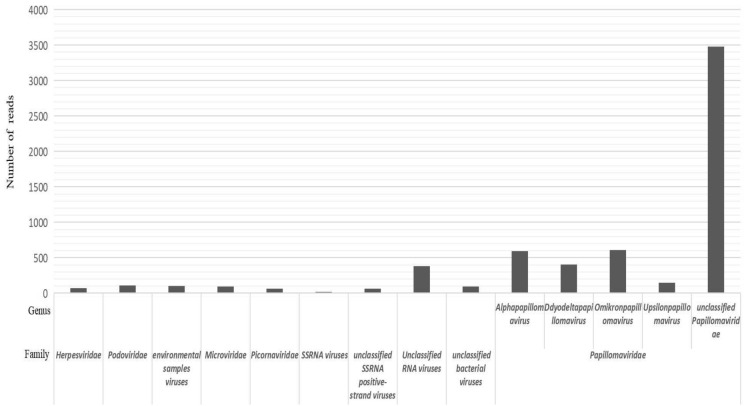
Viral reads of belugas by high-throughput next-generation sequencing.

In total, 28 oral, throat, and anal swab samples from 12 captive belugas (*Delphinapterus leucas*) were acquired and were combined into two pools. We found new viruses for construction and NGS in one of the pools. A total of 1.91 Gb of nucleotide data (19,732,441 valid reads, 100 bp in length) were obtained. Reads that were classified as being from cellular organisms (such as bacteria, archaea, and eukaryotes) and those with no significant similarities to any amino acid (aa) sequences in the non-redundant (NR) nucleotide database were removed. The remaining 7517 reads were best-matched with viral proteins from the NR database, which included the families *Herpesviridae*, *Podoviridae*, *Microviridae*, *Picornaviridae*, *Papillomaviridae*, environmental samples viruses, ssRNA viruses, unclassified + ssRNA viruses, unclassified RNA viruses, and unclassified bacterial viruses (Fig. 1). There were 60 *Picornaviridae*-associated reads in the lane. The reads mapped to the picornaviral genome are shown in Supplementary Fig. S1 online.

### Genome analysis

**Figure 2:**
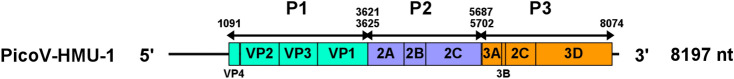
Genome organization of the PicoV-HMU-1 sequence.

**Figure 3:**
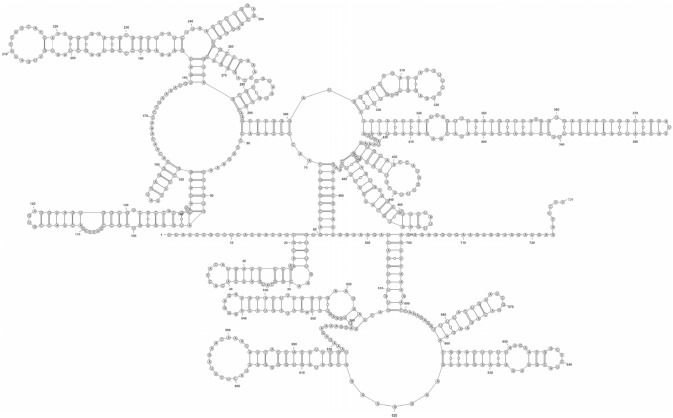
Secondary-structure model for PicoV-HMU-1. The structure was modeled using the aligned RNAalifold server of the ViennaRNA Web Services.

Based on the NGS-generated fragment sequences, primers were designed to obtain the intervening portions of the genome. The terminal sequences were then acquired using a 5' and 3' rapid amplification of cDNA ends (RACE) method. We obtained a picornaviral genome sequence, named the PicoV-HMU-1 strain, which was 8,197 bases in length and with a G + C content of 43.4% after exclusion of the polyadenylated tract. Both the 5'- (1090 bases) and 3'-ends (123 bases) of the genome contained UTRs. The genome contained a ORF of 6,984 bases that encoded a polyprotein precursor of 2328 aa. The sequenced PicoV-HMU-1 genome was predicted to encode the P1 (843 aa), P2 (694 aa), and P3 (791 aa) regions, which potentially encode the capsid proteins VP4, VP2, VP3, and VP1; the non-structural proteins 2A, 2B, and 2C, and 3A, 3B, 3C, and 3D, respectively (Fig. 2). The 5′-UTR of PicoV-HMU-1 was 1090 nt in length. According to the blast analysis, PicoV-HMU-1 shared high sequence identity with Bat picornavirus(HQ595344). We selected 362–1090 bp to analyse the secondary structure by comparison with the complete sequence of HQ595344, and hence we performed the secondary structure prediction shown in Fig. 3.

### Phylogenetic analyses

**Figure 4:**
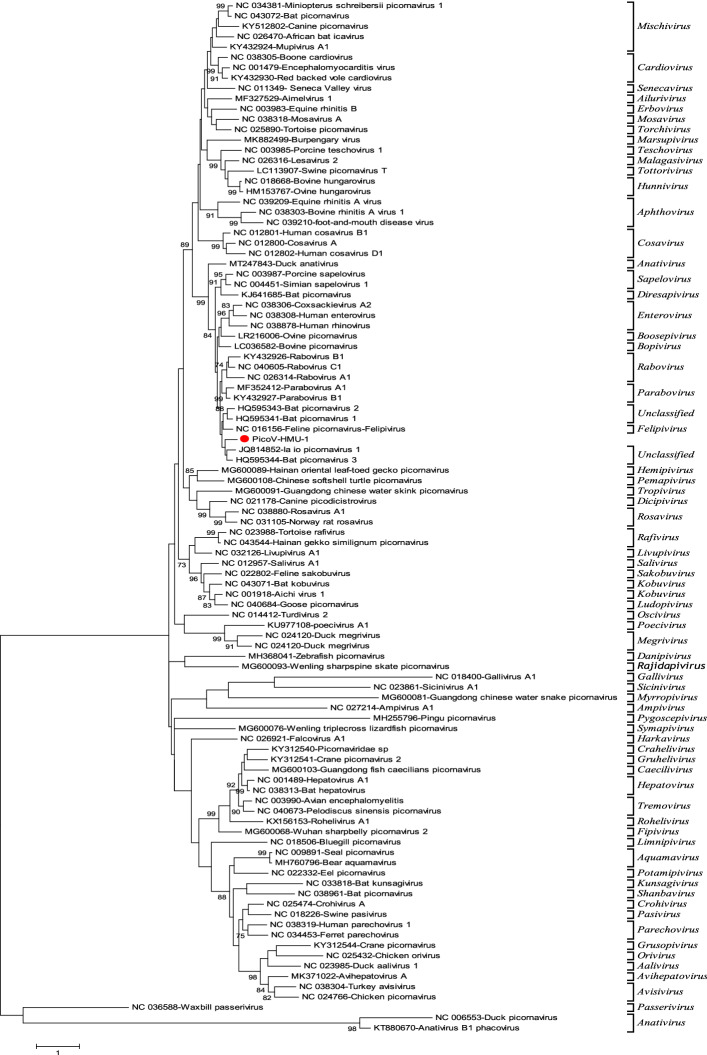
Phylogenetic analyses of the P1 regions of PicoV-HMU-1. Maximum likelihood mtREV with the Freqs (+ F) model, gamma distributed with invariant sites (G + I), and 1000 bootstrap replicates using MEGA 6 software. The virus identified in this study is indicated by the red circle. Bootstrap values are shown on the branches.

**Figure 5:**
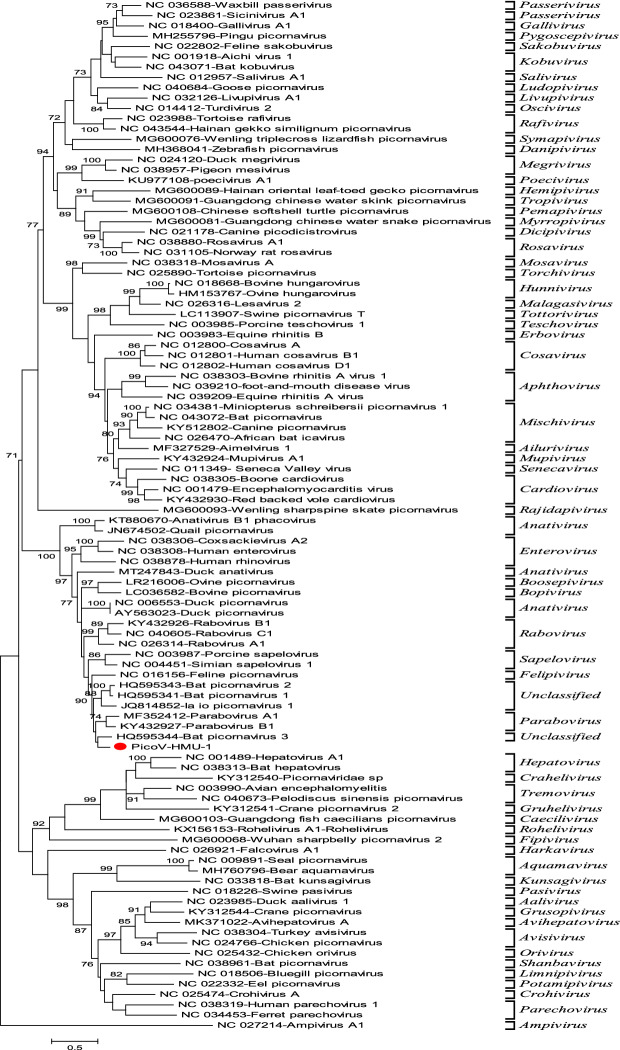
Phylogenetic analyses of the 3D regions of PicoV-HMU-1. Maximum likelihood mtREV with the Freqs (+ F) model, gamma distributed with invariant sites (G + I), and 1000 bootstrap replicates using MEGA 6 software. The virus identified in this study is indicated by the red circle. Bootstrap values are shown on the branches.

The phylogenetic trees were constructed based on the P1 and 3D aa sequences of PicoV-HMU-1 along with representative members of the *Picornaviridae* family and other unclassified viruses. In the P1 trees, PicoV-HMU-1 was close to unclassified bat picornavirus 3 and la_io_picornavirus_1 and formed a monophyletic branch (Fig. 4). In the 3D trees, PicoV-HMU-1 was also related to the unclassified bat picornaviruses 3 and formed an independent cluster (Fig. 5).

### Prevalence of PicoV-HMU-1

All 28 swab samples from 12 captive belugas were tested for PicoV-HMU-1 by nested-polymerase chain reaction (PCR) of the VP1 proteins of PicoV-HMU-1, among which two throat and two anal swab samples were positive (14.29%) (Table 1).

**Table 1:** Prevalence of PicoV-HMU-1 in samples of captive belugas from Qingdao Polar Haichang Ocean Park.

Numbers of captive belugas	Throat swab	Oral swab	Anal swab
1	−	−	−
2	−	Null	−
3	+	Null	+
4	−	−	−
5	+	Null	+
6	−	−	−
7	−	−	−
8	−	Null	−
9	−	Null	−
10	−	Null	−
11	−	Null	−
12	−	Null	−

Samples found to be positive or negative by nested-PCR are indicated by + or – signs, respectively. Null indicates that the sample was not collected.

## Discussion

Previous viral outbreaks in marine mammals (such as morbillivirus infections in European seals and Eastern U.S. dolphins) have temporarily reduced their populations^10^. Morbillivirus infections have also been detected in marine mammals in the Canadian arctic, as evidenced by the presence of neutralizing antibodies in walruses^11^. In 2007, researchers in California identified the first sequence-confirmed picornavirus isolated from ringed seals, which was proposed to represented a member of a new picornavirus genus in the *Picornaviridae* family^12^. In the last year, Australian researchers demonstrated that the novel marine flaviviruses identified in crustaceans were more closely related to the terrestrial vector-borne flaviviruses than classical insect-specific flaviviruses^13^. Although their clinical relevance remains unclear, identification of novel viruses and their respective animal hosts is important for a better understanding of their genetic diversity, evolution, biology, and potential for cross-species transmission and emergence.

In this study, we discovered a new picornavirus within oral, throat, and anal swab samples from white whales using metagenomic technology. Genomic characterization revealed that the structural features of PicoV-HMU-1 are similar to those of other *Picornaviridae* family members, with its genomic length and GC content being within the ranges of those found in picornaviruses. Moreover, the genome organization was similar to that of other picornaviruses, with the characteristic gene order: 5′-VP4-VP2-VP3-VP1-2A-2B-2C-3A-3B-3C^pro^-3D^pol^-3′ (with 3C^pro^ and 3D^pol^ being 3C protease and 3D polymerase, respectively). Picornaviruses encode extensive RNA stem-and-loop structures in the 5′-UTR regions that are critical for viral replication.

The 5′-UTR of PicoV-HMU-1 was 1090 nt in length. According to the Bat picornavirus(HQ595344), We performed the secondary structure prediction with the secondary structure region (362–1090 bp). The secondary structure of the PicoV-HMU-1 sequence is different from that of all currently known IRESs (Fig. 3). At the nucleic acid level, the sequence was barely comparable with that of other known picornaviruses in the National Center for Biotechnology Information nucleotide database. Therefore, we suggest that this secondary structure is of a novel type of IRES. Pair-wise alignment of the aa sequences of PicoV-HMU-1 with those of other picornaviruses revealed that it shared high sequence identity with parabovirus A1 (P1: 48.1%; P2: 35.5%; P3: 57.5%), feline picornavirus (P1: 47.5%; P2: 40.7%; P3: 61.6%), and ovine picornavirus (P1: 45.8%; P2: 35.1%; P3: 53.1%) than with other representative viruses from the recognized genera within *Picornaviridae*. However, its highest sequence identity was with unclassified picornaviruses; bat picornavirus group 1–3 (P1: 450.0–46.9%; P2: 46.3–44.3;%; P3: 67.1–65.6%) (Table 2). Phylogenetic analysis confirmed that PicoV-HMU-1 was related to unclassified bat picornaviruses groups. Therefore, it is likely that these viruses may have shared a common ancestor, and started to evolve independently after terrestrial mammals entered the ocean. Moreover, these results suggest that the host of the novel picornavirus was exclusively the white whale.

**Table 2:** Comparison of genomic features of PicoV-HMU-1 and other species of picornaviruses.

Genus	Name	Accession no	Pairwise amino acid identity (%)		
			P1	P2	P3
Unclassified	Bat_picornavirus_3	HQ595344	50	46.3	67.1
Unclassified	Ia_io_picornavirus_1	JQ814852	48.9	43.5	64.4
*Parabovirus*	Parabovirus_A1	MF352412	48.1	35.5	57.5
Unclassified	Bat_picornavirus_2	HQ595343	48	45.8	67.3
*Felipivirus*	Feline_picornavirus	NC_016156	47.5	40.7	61.6
Unclassified	Bat_picornavirus_1	HQ595341	46.9	44.3	65.6
*Boosepivirus*	Ovine_picornavirus	LR216006	45.8	35.1	53.1
*Bopivirus*	Bovine_picornavirus	LC036582	45.2	41	53.6
*Sapelovirus*	Simian_sapelovirus_1	NC_004451	40.2	30.1	55.3
*Rabovirus*	Rabovirus_A1	NC_026314	38.7	40.6	51.3
*Anativirus*	Duck_picornavirus	NC_006553	37.6	25.5	49.5
*Enterovirus*	Human_enterovirus	NC_038308	35.7	24.6	45.3
*Diresapivirus*	Bat_picornavirus	KJ641685	33.4	26.8	48.6
*Mupivirus*	Mupivirus_A1	KY432924	21.6	15.2	20.5
*Cardiovirus*	Encephalomyocarditis_virus	NC_001479	21	15.3	18.9
*Cosavirus*	Human_cosavirus_B1	NC_012801	20.6	15.2	19.5
*Malagasivirus*	Lesavirus_2	NC_026316	20.5	13.6	21.2
*Mischivirus*	Canine_picornavirus	KY512802	20.1	15.1	19.6
*Senecavirus*	Seneca_Valley_virus	NC_011349	20	14.2	19.7

According to our findings, PicoV-HMU-1 has a lower aa identity and a distinct host species compared with other members of the *Picornaviridae* family. Thus, we propose that the novel PicoV-HMU-1 is a new picornavirus genus. Nevertheless, the role of this picornavirus in the evolution, transmission, and biology of marine mammals warrants further investigation. Thus, it is important to document the existence of novel picornaviruses isolated from marine mammals. Moreover, future studies investigating the evolution, classification, and traceability of mammalian virology should pay increased attention to viruses carried by marine mammals.

## Materials and methods

### Sample collection

The sampling procedures were approved by the Ethics Committee of the Hainan Medical University (Approval number: HMUEC20180059). A total of 28 oral swabs, throat swabs, and swab samples of were collected from 12 captive belugas (*Delphinapterus leucas*) from Qingdao Polar Haichang Ocean Park and Dalian Sun Asia Tourism Holding Co., China, from January to December 2018. The samples were immersed in maintenance medium in virus-sampling tubes (Yocon, Beijing, China), transported to the laboratory within 24 h using cold chain transportation, and stored at − 80 ℃.

### Viral nucleic acid library construction and next-generation sequencing

All 28 samples were combined into two pools based on sampling time and were passed through 0.45 µm filters. The collected filtrates were digested with DNase (Applied Biosystems, Waltham, MA, USA) to remove unprotected nucleic acids. Total RNA was extracted using a QIAamp Viral RNA Mini Kit (Qiagen, Hilden, Germany) according to the manufacturer’s instructions. cDNA was generated using Superscript III Reverse Transcriptase (Invitrogen, Waltham, MA, USA). Amplified viral nucleic acid libraries were analyzed on an HiSeq2500 sequencer (Illumina, San Diego, CA, USA) for a single read of 100 bp in length. The raw sequence reads were filtered using previously described criteria to obtain valid sequences^14^.

### Taxonomic assignment

Sequence similarity-based taxonomic assignments were conducted as previously described^14^. Briefly, each read was evaluated for viral origin by conducting alignments with the non-redundant (NR) nucleotide and protein databases of the National Center for Biotechnology Information, using BLASTn and BLASTx, respectively (Expected value < 10^−5^, F: Filter query sequence, default = T). Taxonomies of aligned-reads with the best BLAST scores (*E*-value < 10^−5^) from all lanes were parsed and exported using the Metagenome Analyzer (MEGAN) 6^15^.

### Genome sequencing

Molecular clues provided by metagenomic analyses were used to classify sequence reads into a virus family or genus using MEGAN 6. Viral RNA was isolated using QIAamp Viral RNA Mini Kit (Qiagen), and cDNA was generated using random primer and Superscript III Reverse Transcriptase (Invitrogen) according to the manufacturer’s instructions. Gene-specific primers were designed based on representative identified reads of the novel picornavirus while ensuring that they covered the partial genomes by nested-PCR amplification and Sanger sequencing. The remaining genomic sequences were obtained by amplification using a genome-walking kit (Takara, Kusatsu, Japan), and the full 5′- and 3′-end sequences were obtained by repeated amplification using a 5′-RACE system version 2.0 combo (Invitrogen) and 3′-Full RACE Core Set with PrimeScript RTase (Takara) according to the manufacturers’ instructions. All primer sequences were based on the newly obtained reads and newly amplified sequences. All primers used are shown in Supplementary Table S1 online.

### Detection of picornavirus

Using the genomic sequences of the viruses obtained by amplification of the ends, specific primers for nested-PCR were designed to screen for picornavirus in the 28 swabs samples collected from 12 captive belugas. PCRs were performed using KOD One PCR Master Mix (Takara), and the first PCR round was primed with outer primers (F: 5′-AGCAGTTACCTTGCCCACG-3′ and R: 5′-TCCCTGCTCGCACCTTG-3′), and the second PCR round was primed with inner primers (F: 5′-CGCCTGAGACTGGTGT-3′ and R: 5′-TTGCCATTGGGTGTAA-3′). PCR product (1 µL) from the first round was used as template for the second round reaction. The thermal cycling conditions for the first round PCR were 98 ℃ for 5 min, followed by 35 cycles at 98 ℃ for 10 s, 50 ℃ for 5 s, 68 ℃ for 5 s, and a final elongation step at 68 ℃ for 1 min; and for the second round PCR were 98 ℃ for 5 min, followed by 35 cycles of 98 ℃ for 10 s, 58 ℃ for 5 s, 68 ℃ for 5 s, and a final elongation step at 68 ℃ for 1 min. Finally, the PCR products were analyzed by 1% agarose gel electrophoresis ultraviolet imaging. Positive samples were determined by the presence of 642 bp amplified products. All PCR products were further verified by Sanger sequencing.

### Genome annotation and phylogenetic analysis

Picornaviruses containing IRES-like sequences in the 5′-UTRs were identified by BLAST searches (http://www.ncbi.nlm.nih.gov/BLAST/) of viral sequences in the GenBank database. Secondary/tertiary structural elements in picornavirus 5′-UTRs were modeled using the aligned RNAalifold server of the ViennaRNA Web Services (http://rna.tbi.univie.ac.at/). MEGA 6.0 (http://www.megasoftware.net)^15^ was used to align the nt sequences and deduce the corresponding aa sequences using the MUSCLE package with default parameters. Phylogenetic trees showing the relationships among picornaviruses based on the amino acid sequences of P1 and 3D were generated using maximum likelihood mtREV with Freqs (+ F) model, gamma distributed with invariant sites (G + I), and 1000 bootstrap replicates. Routine sequence alignments were performed using Clustal Omega, EMBOSS Needle (both available at: http://www.ebi.ac.uk/Tools/), MegAlign and Lasergene (DNAstar, Madison, WI, USA), and T-coffee (http://www.tcoffee.org/) with manual curation. The nt sequences of the genomes and aa sequences of the ORF were deduced by comparison with those of other picornaviruses. The conserved protein families and domains were predicted using Pfam and InterProScan 5 (available at: http://www.ebi.ac.uk/services/proteins). Amino acid identities and genetic distances were calculated using the ML method and were performed using pairwise evolutionary distance calculation as the distance metric.

### Animal rights statement

Animals were treated according to the guidelines of Regulations for the Administration of Laboratory Animals (Decree No. 2 of the State Science and Technology Commission of the People’s Republic of China, 1988). The sampling procedure was approved by the Ethics Committee of the Hainan Medical University.

## Supplementary Information

Supplementary Information.

## Data Availability

The genome sequences of PicoV-HMU-1 are available at GenBank (https://www.ncbi.nlm.nih.gov/genbank/), with accession number: MW883077.
