# Supplementary material for: Identification and genome analysis of a novel picornavirus from captive belugas (Delphinapterus leucas) in China
Source: Sci Rep. 2021 Oct 25;11:21018. doi: 10.1038/s41598-021-00605-y (PMC8549006; doi:10.1038/s41598-021-00605-y)

Identification and genome analysis of a novel picornavirus from captive belugas (*Delphinapterus leucas*) in China

Gaoyu Wang[1,3,4†], Yi Huang[1,3,4†], Weijia Zhang[2†], Ruoyan Peng[1,3,4†], Jun Luo[6], Sisi Liu[7], Shijie Bai[2], Xiaoyuan Hu[1,3,4], Zhiqiang Wu[5], Fan Yang[5], Shu Shen[8], Yun Zhang[1,3,4], Chuanning Tang[1,3,4], Xiuji Cui[1,3,4], Lina Niu[1,3,4] Gang Lu[1,3,4], Songhai Li[2], Fei Deng[8*], Peijun Zhang[2*], Jiang Du[1,3,4*], Feifei Yin[1,3,4*]

Running title: Phylogenetic analysis of a novel Picornavirus

[1]Key Laboratory of Tropical Translational Medicine of Ministry of Education, Hainan Medical University, Haikou 571199, China.

[2]Institute of Deep-sea Science and Engineering, Chinese Academy of Sciences

[3]Hainan Medical University-The University of Hong Kong Joint Laboratory of Tropical Infectious Diseases, Hainan Medical University, Haikou, 571199, China.

[4]Department of Pathogen Biology, Hainan Medical University, Haikou, 571199, China.

[5]NHC Key Laboratory of Systems Biology of Pathogens,Institute of Pathogen Biology, Chinese Academy of Medical Sciences & Peking Union Medical College, Beijing, 100005, China.

[6] Dalian Sun Asia Tourism Holding Co. Ltd., Dalian, 116023, China.

[7] Qingdao Polar Haichang Ocean Park, Qingdao, 266003, China

[8]State Key Laboratory of Virology and National Virus Resource Center, Wuhan Institute of Virology, Chinese Academy of Sciences, Wuhan, China

[†]These authors contributed equally to this work.

[*]These authors contributed equally to this work and are co-senior authors.

[*]Correspondence: yinfeifeiff@163.com (Feifei Yin), dujiangemail@163.com (Jiang Du), pjzhang@idsse.ac.cn (Peijun Zhang)

Supplementary Table S1. Primers used in this study

Primers	Sequences (5' →3')	Position	Amplified products length(bp)
picoM_184F1	GAGAGAGGTGAAGTGGTAGATGAGA	236-2337	2101
picoM_2379R1	CCAAGCTGGATCATTTATCTTACTA		
picoM_236F2	CCTCACGGGCAACACAAGAAGAT		
picoM_2337R2	GACACAACCAGGGGGCACAA		
picoM_2112F1	ACAATTGTGGTTTGATAAATCTGAT	2164-4277	2113
picoM_4370R1	CAAAATGCTTAGGATCTGGGGGTA		
picoM_2164F2	TACAGAATGTTTACTTATTTGCGGT		
picoM_4277R2	GACTTTCCGGTCCCTGGTGTTC		
picoM_4010F1	CCCTTTTGATGGAACATATAGATAA	4043-5608	1565
picoM_5664R1	CCCTCCACTTTCACCTTGAGTTATT		
picoM_4043F2	CAGCAAGAGGGAAATATTCTGAAGA		
picoM_5608R2	CACCATAACCATTTGCTCCATCA		
PicoM_5612F1	TGCGAAGTAGTGAGGCGGATAA	5681-6180	499
PicoM_6704R1	GTTGGGACGGGTAGTGGGAAA		
PicoM_5681F2	GACACCGTCAACATCATCGCTCT		
PicoM_6180R2	GAAAAGATCTATCTGAAGCCGTATT		
PicoM_563GSP1	GCTCACCCTCATCCTT	37-542	505
PicoM_296GSP2	CTGGTTCTGACAAGGCATCT		
PicoM_42GSP3	GCACCTCCATAACCACCAT		
PicoM37_5060Outer	GATCAGTGGCTTCTTCTTCC	5252-8179	2927
PicoM37_5252Inner	GAGGGTGATTGCATGTTAGTG		
PicoM_37_4845Outer	CATCCATAGCAGGCTTCATT	5245-8179	2934
PicoM_37_5245Inner	TCAGAGGGAGGGTGATTGC		
PicoV-F1-2788	AGCAGTTACCTTGCCCACG	2847-3489	642
PicoV-R1-3614	TCCCTGCTCGCACCTTG		
PicoV-F2-2847	CGCCTGAGACTGGTGT		
PicoV-R2-3489	TTGCCATTGGGTGTAA		

Supplementary Fig. S1. Sixty *Picornaviridae*-associated reads mapped to the picornaviral genome.

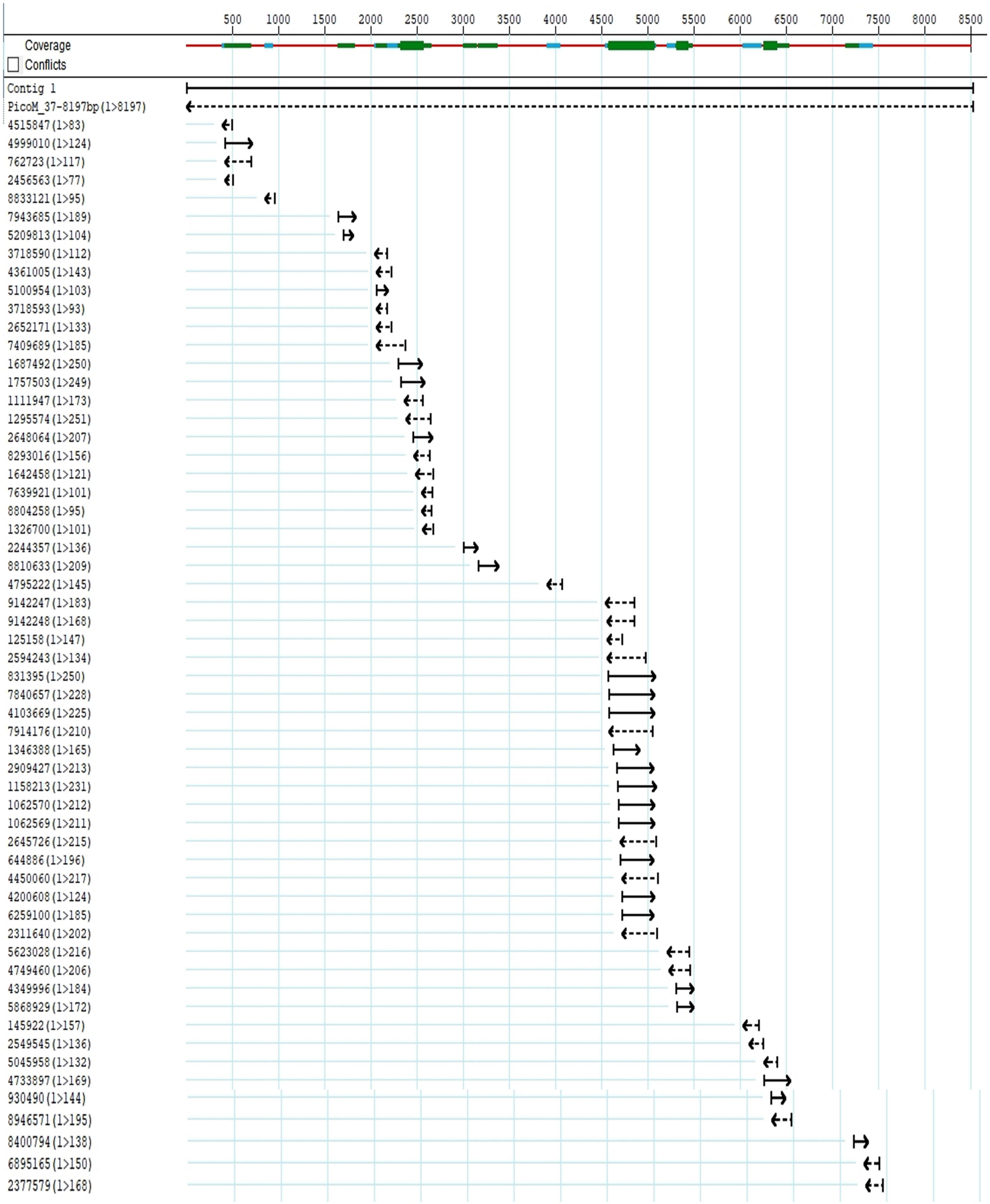

Supplement: Supplementary file 1 — Supplementary Information. [file 41598_2021_605_MOESM1_ESM.pdf]
